# Experiences of nurses on the critical shortage of medical equipment at a rural district hospital in South Africa: a qualitative study

**DOI:** 10.11604/pamj.2017.28.100.11641

**Published:** 2017-09-29

**Authors:** Merriam Bautile Moyimane, Sogo France Matlala, Mokoko Percy Kekana

**Affiliations:** 1 Department of Public Health, University of Limpopo, Private bag X1106, Sovenga, 0727, South Africa

**Keywords:** Critical equipment shortage, District hospital, experiences

## Abstract

**Introduction:**

Medical equipment is an essential health intervention tool used by nurses for prevention, diagnosis and treatment of disease and for rehabilitation of patients. However, access to functioning medical equipment is a challenge in low- and middle-income countries. The World Health Organization estimated that 50 to 80 percent of medical equipment in developing countries is not working, creating a barrier to the ability of the health system to deliver health services to patients. This study explored and described the lived experiences of nurses working at a district hospital with a critical shortage of medical equipment.

**Methods:**

A qualitative, exploratory, phenomenological and descriptive study design was used. A purposive sampling was used to select participants and due to saturation of data 14 nurses participated in the study. Research ethics were observed. Data was collected through semi-structured interviews using an interview guide. Interviews were audio-taped and field notes were taken. Voice recordings were transcribed verbatim and Tesch’s open coding method was used for data analysis. Findings were confirmed by an independent coder.

**Results:**

Critical shortage of medical equipment at the hospital occurred in the form of unavailability of equipment, low quality and poor maintenance of the few that were available. Shortage impacted negatively on nursing care, nursing profession and the hospital.

**Conclusion:**

Nurses should be provided with functional medical equipment in order to provide quality nursing care. Management, leadership and governance structures should be strengthened to ensure that procurement and maintenance plans for medical equipment are developed and implemented.

## Introduction

Medical equipment is an important component of a health system and is a tool used by nurses to prevent, diagnose, monitor and treat diseases as well as during rehabilitation after disease or injury. It can be in the form of a machine, instrument, appliance, software or material intended by the manufacturer to be used alone or in combination with other devices [1]. Medical equipment has a lifecycle requiring calibration, maintenance, rweepair, user training and finally retirement [2]. A responsive health system guarantees communities equitable access to essential medical equipment of assured quality, safety and cost effectiveness.

Shortage of medical equipment, either due to unavailability or non-functioning, is a barrier to the ability of the health system to deliver quality health services. The World Health Organization estimates that between 50 to 80 percent of medical equipment in developing countries is not functioning and those countries lack technology assessment systems and regulatory controls to prevent importation of inferior medical equipment. These make the countries exposed to dishonest market practices that put patient’s lives at risk [3].

One of the authors of this study worked in a rural district hospital for more than twenty years as professional nurse and nursing manager. She experienced shortages of medical equipment and also received complaints from other nurses through staff meetings, reports and staff exit interviews. Some of the medical equipment were old and obsolete while others were broken. The budget for maintenance and repairs was centralised at a bigger hospital in the province making procurement a lengthy process. A visit to this district hospital by a parliamentary committee on health in 2012 declared the shortage of medical equipment as critical [4]. Critical shortage refers to a situation where resources required to sustain human life, prevent permanent disability, or stabilise a person experiencing a medical emergency are depleted and alternative methods of obtaining them have been exhausted such that the remaining resources will not make it possible for the hospital to treat patients according to the national core standards [5].

It is against this background that the study was conducted. This paper describes the design of the study, methods used to collect and analyse data and discusses the findings in light of relevant literature. A conclusion and recommendations are made.

## Methods

**Study design:** The study used a qualitative, exploratory, descriptive and phenomenological design. Qualitative phenomenological studies focus on the meaning and understanding of experiences as lived by participants [6]. A descriptive research design involves identification of problems within a certain practice and justification of that practice. This assists researchers to determine what other professionals in similar situations are doing [7].

### Study site

The study was conducted at a large district hospital in Mpumalanga province of South Africa. The province has 33 hospitals and 23 of them are district hospitals [8]. This district hospital had 423 approved beds and 329 of them were in use. It had eleven wards, including a theatre, high-care unit, maternity, an outpatient department, casualty, paediatric, medical, surgical, mental health, TB and step-down wards, as well as dental, x-ray and rehabilitation units. There were 14 primary health care clinics and one community health centre within the catchment area of the hospital. The hospital served a population of 433 163 and is situated in a rural area.

### Collection of information and participants

Participants were employed as professional nurses, staff nurses and nursing assistants according to nursing categories of the South African Nursing Council. We used a purposive sampling method to select participants based on their experiences of using medical equipment in their work. We conducted semi-structured interviews, using an interview guide, until saturation of data was reached. Saturation of data occurs when an interviewer realises that there is repetition of revealed information and confirmation of previously confirmed information [9]. We conducted all interviews in English from March to May 2015, each of which lasted for between 45 and 60 minutes.

The central question was *“What are your experiences regarding critical shortage of medical equipment in this hospital?”*. During the interviews, we used deliberate probing and clarifying as well as summarising as technigues to increase clarity of the responses [10]. Responses were captured though an audiotape and we also collected field notes. We chose semi-structured interview as the strategy that would cause minimal disturbance to nursing care as nurses were interviewed individually as they became available.

### Data analysis

Data analysis started during the interviews when the interviewer was listening attentively to responses to identify repetition of things already said by other participants interviewed earlier. Once all the interviews were completed, verbatim transcriptions of the voice recordings followed. We independently used Tesch’s open coding method of data analysis [11] to analyse transcripts and field notes taken during data collection and at the end compared our findings. We then submitted the transcripts together with field notes to an independent coder for verification and later compared the findings of the independent coder with the codes we found. We met with the independent coder to discuss and reach agreement on the codes.

### Ethical considerations

Ethical clearance was obtained from Research Ethics Committee of the University of Limpopo (MREC/HS/321/2014: PG) while permission to access participants was obtained from Mpumalanga Department of Health and management of the district hospital. We respected the rights of participants to autonomy and self-determination by obtaining their informed consent and allowing them the right to withdraw from the study without punishment [6]. Permission to collect field notes and to use audio recorder was obtained from participants. We conducted interviews in an office to ensure privacy and kept data in a locked place accessible to the researchers only. Furthermore, we asked only those questions relevant to the aims and objectives of the study. We advised participants not to mention their names or the names of other persons during the interviews to prevent linking of data with participant’s identities [12].

## Results

Fourteen nurses working in gynaecology, operating theatre, casualty, paediatric / children's, medical, surgery and orthopaedic wards participated in the study. Others were working in quality assurance, occupational health and safety, mental health units and the nursing school (Table 1). Two themes and six sub-themes emerged (Table 2).

**Table 1:** Demographic profile of participants

Nursing category	Female	Male	Number
Professional nurse	5	5	10
Staff nurse	2	0	2
Nursing Assistant	1	1	2
Total	8	6	14

**Table 2:** Themes and sub-themes

Themes	Sub-themes
Encounters with shortages of medical equipment	Unavailability of medical equipment Low quality medical equipment Poor maintenance of medical equipment
Consequence of the shortages of medical equipment	Impact on patient care and service delivery Impact on nurses and nursing profession Legal implications for the hospital

### Encounters with shortages of medical equipment

Critical shortages of medical equipment appeared in the form of unavailability of equipment accompanied by low quality and poor maintenance of the few that were available.

#### Unavailability of medical equipment

Nurses related their dissatisfaction about the unavailability of basic diagnostic, resuscitation and monitoring equipment. A professional nurse working in theatre said: *“So the lack of the C-arm machine is of great disadvantage to theatre when the operation of such a magnitude of bone should be done”*. Another professional nurse working in casualty said: *“You want to resuscitate a patient and you don’t even have a functional ventilator, and you just bag the patient until Emergency Medical Services arrive or until the patient is taken to the referral hospital”*. A nursing assistant working in a medical ward said: *“We have only one glucometer, sometimes the batteries get finished, we have…. to dig out of our own pockets to buy batteries’’*.

#### Low quality medical equipment

Some nurses complained about failure of medical equipment to meet its performance specifications or to perform as intended due to its low quality. A professional nurse working as Quality Assurance Coordinator said:*“What we find is that the medical equipment that we receive happen to be of poor quality most of the time……you buy them… they quickly fail, and you don’t have to replace them. I can give an example of oxygen gauges, we do procure enough, but most of them will fail in no time. Another example of the monitors……we do buy them but some of them will give false readings again within a short space of time”*. Another professional nurse working as a trauma nurse added: *“One patient nearly bled to death because of lack of a tourniquet, we had one which was not functioning well. You want to provide service but the things that you are supposed to use to provide service…. they are not there…. or they are there but not functional”*.

#### Poor maintenance of medical equipment

Nurses expressed challenges around medical equipment that is not maintained, replaced or repaired. This was attributed to the fact that there was no service plan for medical equipment. A professional nurse working as Occupational Health and Safety Nurse said: *“There is no service plan for the equipment. They use it for a longer time without service then it gives them a problem in functioning because there is no service”*. Another professional nurse working in theatre added: *“We have an orthopaedic drill which has been long procured and is not in a good working condition, it takes time to drill a bone, some of the drill bits are blunt so it actually take more time than necessary to drill a bone……and need more force to drill that bone…….and sometimes they get broken because they are old”*.

### Consequence of the critical shortages of medical equipment

Some nurses mentioned that challenges of medical equipment shortages affected patient care and service delivery negatively leading to serious consequences to the image of the hospital and the nursing profession. Medical equipment shortage was perceived as responsible for prolonging stay of patients in the hospital, resulting in prolonged procedures for referral of patients. This was perceived as unfair treatment of patients and substandard nursing care.

#### Impact on patient care and service delivery

A professional nurse working in children’s ward said: “Service delivery is severely compromised without proper functional medical equipment”. Another professional nurse from a medical ward added: “The shortage of medical equipment is so big to an extend that we sometimes don’t do some of the nursing care as far as the quality nursing care is concerned.

#### Impact on nurses and nursing profession

Nurses showed emotional reactions through feelings of self-blame and guilt, feelings of being discouraged, feelings of frustration and feelings of demotivation. One professional nurse working in a surgical ward remarked: *“It affects me badly because as a trained orthopaedic nurse I know how to do the correct things, so if I’m doing things the other way round I feel demoralised and I feel so useless to my patients”*. Another professional nurse from an orthopaedic ward stated: *“In some of the equipment failures…. the results, the outcome… it’s eh… deformities, every time when you meet the patient with deformity maybe short leg discrepancy the patient can’t walk well, you blame yourself and say I would have done something to this patient but because you didn’t have proper equipment……there will be that sense of guilt at times to say I could have done better if I had this and that or maybe I could not have given this one but the other one, so ja.. obviously it comes with an elements of frustration and some form of guilt to say maybe I made the wrong decision”*. A nursing assistant working in a medical ward indicated: *“It affects us and we also feel the pain of our patients”. A staff nurse working in a surgical ward complained: “We keep blaming ourselves and this is haunting us. We end up developing stress related conditions due to failure of the hospital to supply us with the required medical equipment”.*

Some nurses were seriously concerned about loss of skills and knowledge they acquired from nursing schools and universities. The situation may contribute to poor staff retention and knowledge deficits. A professional nurse from gynaecology ward said: *“I find that my profession is a little bit shaken….or little bit lacking as far as I have been groomed or I have been taught…..where I got my knowledge, so somehow my profession…..professionally I feel I am not developing or well maturing because I should be utilising the skills…..given the appropriate equipment to execute those skills”.*

#### Legal implications for the hospital

Some nurses expressed a concern that the shortage of medical equipment may lead to negligence, malpractice and even patient deaths which will result in legal actions taken against the hospital. A professional nurse working as Quality Assurance Coordinator said: *“Patient safety is at risk…… that is the core…. that is the biggest implication because when you look at patient safety you really need medical equipment for you to actually guarantee patient safety”*. A staff nurse working in a surgical ward said: *“Failure to monitor patients adequately leads to litigations and disciplinary hearing by the South African Nursing Council which contributes to low morale and working in fear. Shortage of essential medical equipment compromise patients’ life and leads to poor diagnosis of patients. Sometimes patients lose their lives as a result of this problem. Sometimes referral of patients for further management is delayed”.*

#### Impact on training of nurses

The district hospital is accredited as training institution for nursing, medical and allied students. Nurses expressed concerns about inadequate and irrelevant equipment for demonstrating nursing procedures to student nurses, for example incomplete maternity delivery packs and lack of vulva swabbing packs to care for a woman after delivery. A professional nurse working as nursing school lecturer complained: *“As a lecturer dealing with obstetric and new born care, you find that delivery packs are not up to date…. the equipment is not really there. When you have to teach students it gives you a problem, you don’t have adequate of the relevant equipment to use to demonstrate to them, when they reach the clinical area in the wards they don’t have the same experience as they were taught, so you find that there is that challenge… the students don’t really do what they are being taught.* Another nursing school lecturer complained that some of their products may not be able to use certain medical equipment which they were never exposed to during training by saying: *“The students that we are training are not all from our hospital, some are from other hospitals where you find that when they reach their hospital every equipment may be there….. but they are not going to be able to use it properly……”*.

## Discussion

Public hospitals in South Africa experience varying shortages of medical equipment and in general the country scored below 50% in a national health care facilities baseline audit conducted in 2012 [13, 14]. The shortage is higher in rural hospitals than in urban hospitals like in other countries [15-17]. Poor maintenance and repair as well as limited financial resources are responsible for the shortages. Medical equipment maintenance refers to regular servicing and prompt repair of broken equipment to keep it in best possible working condition. A maintenance plan requires sufficient planning, management and implementation which are controlled by the financial, physical and human resources available in a country [2]. Availability of sufficient and well-functioning medical equipment is therefore a challenge to low- and middle-income countries with limited resources [18].

The National Core Standards of Health Establishments in South Africa [19] requires that medical equipment be maintained according to manufacturers’ requirements to keep the equipment reliable, safe and available for use when required for diagnostics, treatment and monitoring of patients. A maintenance plan prolongs the life of medical equipment and minimises the cost associated with buying new equipment. Medical equipment defects and failures are very common in hospitals where there are poor maintenance plans and can lead to injury or death [20]. Hospitals have a responsibility to regularly maintain medical equipment in use to avoid equipment malfunctioning or failure. Nurses and patients rely on medical equipment for proper diagnosis, monitoring, prompt treatment and patient safety [21]. A responsive health system should deliver quality services to all people when and where they need health services. A study conducted in rural Tanzania found that shortages of medical equipment compromised the health systems’ ability to reduce maternal and neonatal mortality [22].

The South African Nursing Council’s objective of clinical practice for student nurses is to provide them with meaningful learning opportunities in all areas of clinical placement to ensure that, on completion of training, nurses are able to provide quality nursing care [23]. Clinical teaching is the means by which student nurses learn the integration of theoretical knowledge and practical skills [24]. Nurses in the current study perceived lack of adequate and relevant teaching materials in the form of medical equipment as having adverse impact on the quality of clinical teaching; it made clinical teaching difficult, impaired effective teaching and learning of best practices and led to poor teaching outcomes. Due to a lack of medical equipment, nurses who were clinical teachers often improvised during clinical teaching, as such, student nurses were not taught ideal nursing procedures. Training of nurses requires integration of theory and practice in hospitals where student nurses interact with patients and their families and the community [25].

Clinical practice provides student nurses with opportunities to achieve professional competence and to apply concepts learned in class. Students get to practice skills learned in the skills laboratory and interact with patients, families and other healthcare workers. A positive and supportive clinical practice setting should influence the integration of theory and practice [26]. Poorly trained nurses will slow down the multidisciplinary health team’s effectiveness to provide quality patient care. As the performance of hospitals depends on the knowledge, skills and motivation of nurses and other multidisciplinary health team members, management should provide working conditions that support nursing performance. Although nurses are an important asset of the health system, improving their productivity and performance remains a challenge for most African countries [27]. In a study conducted in Uganda, nurses expressed concerns of forgetting what they had been taught at nursing schools as some medical equipment was not working or not available to use.

The situation projected a bad image of the nursing profession to the community leading to nurses not being respected by community members anymore, as such they were prompted to leave the hospital. In some cases, nurses were reluctant to go to work at a hospital with shortage of medical equipment where they will face patients and relatives without the necessary equipment to provide nursing care [28]. In South Africa, failure to provide quality nursing care can result in nurses, the hospital or the minister of health being sued by patients [14]. Hence the concern of nurses with the critical shortage of medical equipment at the district hospital.

## Conclusion

Medical equipment is a necessary tool for nurses to provide quality nursing care. Shortage of medical equipment has a negative impact on patients, the hospital and nursing profession, as such, it is a barrier for the health system to function. Critical shortages of medical equipment in the hospital resulted from malfunctioning equipment, poor maintenance and unavailability of equipment caused by budgetary constraints. Proper management, leadership and governance is required to develop and implement procurement, maintenance and quality control plans. These will help to prolong equipment lifespan and reduce potential risks due to frequent breakdowns.

### What is known about this topic

- Medical equipment is an indispensable tool for nurses to provide nursing care;
- Medical equipment needs regular maintenance and repairs to remain functional;
- Most African countries experience shortages of medical equipment.

### What this study adds

- There is a district hospital in South Africa where shortage of medical equipment was confirmed as critical;
- Shortage of medical equipment frustrates nurses in their determination to deliver quality nursing care;
- Shortage of medical equipment affect the training of nurses negatively.

## Competing interests

The authors declare no competing interests.
